# Economic versus technical efficiency in using ASM combined with fungicides to elicit wheat varieties with different disease susceptibilities

**DOI:** 10.1016/j.heliyon.2023.e17012

**Published:** 2023-06-09

**Authors:** Lucas Vinicius Dallacorte, Marco Antonio Bosse, Diogo Capelin, Marcos Vily Paladini, Felipe Cattani, Mateus Batista Remor, José Donizetti de Lima, Anelise Tessari Perboni, José Abramo Marchese

**Affiliations:** a Agronomy Department, Federal University of Technology – Paraná, Pato Branco, PR, Brazil; b Syngenta Crop Protection, São Paulo, SP, Brazil; c Bioprocess and Biotechnology Engineering Department, Federal University of Technology – Paraná, Dois Vizinhos, PR, Brazil

**Keywords:** *Triticum aestivum*, Fungal diseases, Elicitors, Sustainable agriculture, Economic evaluation, Plant immunity

## Abstract

Despite the positive results of using elicitors to induce resistance against plant diseases, some factors have inhibited the popularization of their use in agriculture. There is an energetic cost related to the elicitors' induced response which can cause undesired effects on growth under low-pressure disease conditions. Elicitors can create phytotoxicity and show high variation in their efficiency between different genotypes within the same crop; in addition, the positive results related to the induced resistance may not repeat in field treatments, adding to the possibility that they are not economically viable. Thus, we carried out two experiments to investigate the technical and economic efficiency of acibenzolar-S-methyl (ASM) and its association with fungicides in the control of leaf diseases of susceptible and resistant wheat varieties, and as how it reflects on the photosynthetic and production performance of wheat. This study showed the limitations of incorporating ASM into foliar fungal disease control in economic terms. However, it was evident that ASM effectively induced plant resistance against Leaf Rust and Powdery Mildew in the field and can be considered a sustainable option for wheat cultivation. Even though its association with chemical control was not the best economic strategy the use of ASM is a tool that can be incorporated into wheat cultivation to minimize the emergence of fungicide-resistant pathogens due to the diversification of modes of action employed and reduce the toxic residue deposition to the environment and human health.

## 1 Introduction

Wheat (*Triticum aestivum* L.) is the second most produced cereal in the world, with 756.949 million tons produced on 217.898 million hectares (ha) in 2020 [1]. It is one of the primary food sources in the world and serves as a raw material in food manufacturing, where it is used to produce items such as bread, cakes, cookies, and pasta, with a constant increase in demand [2,3]. This cereal has been cultivated in Brazil for over 100 years, with constant yield gains, highlighting the state of Rio Grande do Sul as the most prominent producer [4,5].

The crop is affected by many pathogens that can cause plant diseases in the field. In southern Brazil, the most common diseases in wheat are Powdery Mildew (*Blumeria graminis* (DC) Speer f. sp. tritici Em. Marchal), Leaf Rust (*Puccinia recondita* Rob. ex Desm. f. sp. tritici), Yellow Spot (*Drechslera tritici-repentis* (Died) Dresch), Brown Spot (*Bipolaris sorokiniana* (Sacc.) Shoem), and Fusarium Head Blight (*Gibberella zeae* Schw.) [6].

Domesticated and bred plants have higher crop yields but smaller resistance compared to wild plants, resulting in crops susceptible to diseases that cause significant yield losses for a large number of crops [7] and waste resources such as the energy and water invested in crop growth [8]. While the zero tolerance for disease philosophy is often not achievable, technology favoring the complex plant immune system is a viable alternative [9].

Wheat diseases affect the leaf structure, reducing absorption effectiveness and usage of solar radiation in the affected areas [10]. Plants must allocate resources to combat the growth and multiplication of pathogens responsible for the infection, which involves the action of effector molecules - virulence factor [11]. The pathogen propagation in the plants will depend on the plant's defense mechanism, which often is not activated or activated late. Therefore, studies have investigated the activation mechanisms that will make crops more tolerant to pathogens [12].

Due to the rapid evolution of wheat pathogens, the genetic resistance of crop varieties has lost its efficiency as cycles pass [8,13]. Therefore, more than the use of resistant varieties is required. Wheat pathogens can evolve by migration, mutation, or virulence gene recombination; the pathogens responsible for the Leaf Rust and Powdery Mildew presented this behavior more frequently [14]. The genetic background effect should also be considered, which determines that the expression of a disease resistance gene can vary amongst wheat cultivars [8]. Besides, the search and selection of genetic materials focused on high yield performance reflected the decline of genetic diversity, making crop diseases a threat to the global wheat supply [6].

Genetic resistance, healthy seeds, seed treatment, crop rotation, biological control, and chemical control are methods used in wheat disease management, the latter being significant to the production costs [15,16]. Despite the high investment required, chemical control is still the most used control method in wheat; however, the indiscriminate usage of chemicals associated with this method reflects in environmental damage, human health issues, and the increase of selection pressure over diseases and pests [17].

One of the biggest challenges for crop management is the development of eco-friendly control alternatives aiming at more sustainable agriculture. Alternative products such as elicitors (natural or synthetic substances that mimic pathogen/microbes/herbivore-associated molecular patterns (PAMPs/MAMPs/HAMPs) are among the potential options for reducing the use of chemical fungicides. Elicitors are widely studied disease control alternatives, serving as measures to reduce crop management environmental impact [18,19]. These products do not directly inhibit pathogens but induce a faster and stronger activation of the defense responses in the host plants.

The term “plant immunity” refers to its resistance to diseases at a cellular level [16]. The plant's defense mechanism can be divided into two branches. The first relates to the Pattern Recognition Receptors (PRRs), which recognize molecular patterns (PAMPs/MAMPs/HAMPs). This type of immunity is a basal defense response called Pattern Triggered Immunity (PTI) [20]. The second branch of a plant's immune system is realized by resistance proteins (Proteins R) that recognize pathogen-specific effectors (Avr proteins) and activate the plant's defense mechanism [21]. This type of resistance is denominated as Effector Triggered Immunity (ETI). While on PTI and ETI, the defense signs are transmitted from receptors responsible for the pathogen's resistance [22]. Regarding ETI and PTI-induced signaling pathways, the three hormonal pathways that stand out involve salicylic acid (SA), jasmonic acid, and ethylene [23].

Any interaction between plants and phytopathogens involves the generation of various chemical molecules critical for the activation of their defense mechanism. One of the chemicals, SA induces systemic acquired resistance (SAR) in plants. The activation of SAR provides broad-spectrum resistance against related or unrelated pathogens. There has been considerable progress in the biochemical and molecular understanding of SAR activation in various plants [11]. In addition, several chemicals, including SA and its most studied analog, acibenzolar-S-methyl (ASM), are known to provide a direct or indirect defense against pathogens when applied to plants [11,21].

The ASM was the first commercial salicylic acid synthetic analog to promote systemic acquired resistance in plants [24,25]. Several studies describe ASM's efficiency in inducing disease resistance in cultivated species [13,[24], [25], [26], [27], [28], [29]].

Despite the positive results of using elicitors to induce resistance against diseases in plants, some factors have inhibited the popularization of their use in agriculture. According to Refs. [11,30] there is an energetic cost related to the elicitors' induced response which can cause undesired effects on growth under low-pressure disease conditions. The researchers also highlighted that elicitors could create phytotoxicity and show high variance in their efficiency amongst different genotypes within the same crop; in addition, the positive results related to the induced resistance may not repeat in field treatments, adding to the possibility that they are not economically viable.

Thus, we carried out two experiments to investigate the technical and economic efficiency of ASM and its association with fungicides in the control of susceptible and resistant wheat varieties’ leaf diseases, and how it reflects on the photosynthetic and production performance of wheat. The first experiment investigated the effects of ASM associated with standard fungicides used in wheat cultivation for cultivars that are susceptible to diseases. As the results showed that cultivars susceptible to diseases benefited the most from the inducer, a second experiment was conducted to investigate both the use of ASM alone and ASM associated with other fungicides approved for disease control in wheat.

## 2 Material and methods

Two experiments were conducted in the field in two different locations to evaluate the acibenzolar-S-methyl (ASM; Bion 500 WG – dispersible granules; Syngenta Crop Protection, Brazil) efficiency when associated with fungicides on wheat and applied in different development stages as per [31] phenological growth scale. The environmental data from both sites are shown in Supplementary Fig. 1.

The first experiment investigated the effects of applying ASM combined with standard wheat fungicides to two cultivars with different disease susceptibility. The second experiment utilized only one cultivar as it investigated the use of ASM isolated and ASM combined with different fungicides currently used to control wheat diseases. The infestation of plants by diseases occurred naturally.

### 2.1 Experiment 1

The experiment was conducted in the Experimental Area of the Federal Technological University of Paraná, in Pato Branco, Paraná, Brazil (−26°07′S and −52°41′ W - 760 m high). The climate classification according to Köppen-Geiger of this area is humid mesothermal subtropical with hot summer (Cfa) [32].

The experimental design used was the Randomized Complete Block Design with parcels subdivided into four replications. Each variety's main parcel was subdivided exclusively for fungicide applications (control group) and two ASM doses associated with a fungicide. The sub-parcels (1.6 m length) added up to 4 sub-parcels per treatment and were composed of 9 lines, each spaced in 0.2 m of length, with a total of 8 m^2^.

Two wheat varieties were used: (i) Safira (developed by OR Seeds and Biotrigo Genética, in 2003), which is moderately resistant to Leaf Rust and Powdery Mildew and moderately susceptible to leaf spots and Fusarium Head Blight; and (ii) BRS Tangará (registered by EMBRAPA - Empresa Brasileira de Pesquisa Agropecuária, in 2007), which is resistant to Leaf Rust and Powdery Mildew and moderate resistant to Brown Spot and Yellow Spot. The seedling was realized with a parcel seedling machine on June 10, with a density of 70 seeds per linear meter. 40 kg ha^−1^ of nitrogen was used as base fertilizer, complemented by 45 kg N ha^−1^ during tillering (Zadoks - 2.4).

The application of treatments happened in two stages (tillering and grain filling) with a CO_2_-pressurized backpack sprayer, utilizing two doses of ASM (15 g and 25 g ha^−1^). At tillering (Zadoks - 2.4), the two doses of ASM were applied along with fungicide Propiconazole (250 g L^−1^) in a dose of 0.3 L ha^−1^ + mineral oil at 0.5 L ha^−1^ and a spray volume of 300 L ha^−1^. The second application of both ASM doses was in the grain filling period (Zadoks - 4.5), applied along with fungicide Azoxistrobin (800 g L^−1^) + Ciproconazole (80 g L^−1^) in a dose of 0.3 L ha^−1^ + mineral oil at 0.5 L ha^−1^ and a spray volume of 200 L ha^−1^.

An analysis of incidence and severity of Powdery Mildew (*Blumeria graminis* f.sp. tritici), Leaf Rust (*Puccinia recondita* Rob. ex Desm. f. sp. tritici), Yellow Spot (*Drechslera tritici-repentis* (Died) Dresch), and Brown Spot (*Bipolaris sorokiniana* (Sacc.) Shoem), was conducted in 15 plants (flag leaf and on the leaf immediately below it) per sub parcel when in anthesis phase (Zadoks - 6.1). The incidence analysis was conducted by separating 30 leaves into two groups, one with leaves displaying disease symptoms and a second group without disease symptoms. The same 30 leaves were used to diagnose all diseases related to the experiment.

Only the leaves with lesions larger than 2 mm were considered for disease incidence. The Incidence calculation (%) was based on dividing the number of diseased leaves by the total number of leaves sampled, multiplied by 100 to express the result as a percentage of diseased plants. Disease severity was determined on each leaf by analyzing symptoms using a diagram scale with grades varying from 0 to 100%. The overall severity of the sample (S%) was calculated by averaging the severity scores of the 30 leaves analyzed. Our scales were adapted from Refs. [33,34].

Gas exchanges were analyzed on flag leaf in 5 plants per sub parcel during the first awns visible (Zadoks - 4.9) and grain filling, early milk (Zadoks - 7.3) using an infrared red gas analyzer (IRGA 6400XT, LI-COR - USA). The measurements were taken from the middle region of the fully expanded leaves and completely exposed to solar radiation from 9 to 10:30 a.m. The following characteristics were measured: net photosynthetic rate of CO_2_ (*P*_*n*_, μmol m^−2^ s^−1^); water use efficiency (WUE, μmol mmol^−1^); stomatal conductance (*gs*, mol m^−2^ s^−1^); intercellular CO_2_ concentration (*C*_*i*_, mmol m^−2^ s^−1^), transpiration rate (*E*, mmol m^−2^ s^−1^) and carboxylation efficiency (CE), calculated by the division of *P*_*n*_/*C*_*i*_.

Harvesting of 1 m of each one of the 7 central lines of each sub parcel was used to determine the steam mass (SM, g m^−2^), leaf mass (LM, g m^−2^), the number of spikes m^−2^ (NSP), grains per spike (GPS), the mass of thousand grains (MTG, g) e grain yield (GY, kg ha^−1^).

### 2.2 Experiment 2

Experiment number 2 was conducted in the Experimental Station of the University of Passo Fundo, in Passo Fundo, State of Rio Grande do Sul, Brazil (−28.13′S and −52.23′W, 697 m high); the climate classification according to Köppen-Geiger is the humid subtropical climate (Cfa) [32]. The seedling happened on July 4, with a density of 70 seeds per linear meter, utilizing the variety TBIO Sintonia (registered by Biotrigo Genética, in 2013); this variety is susceptible to Powdery Mildew and Leaf Rust. The base fertilization happened with 15, 102, and 60 kg ha^−1^ of nitrogen, phosphorus, and potassium, respectively. To complement the base fertilization, 90 kg ha^−1^ of nitrogen was applied, divided into two applications.

The experimental delineation was a completely random block, with 8 treatments (Table 1) and 4 replicates. Each plot consisted of 11 rows spaced at 0.17 m, with 3.0 m long. Altogether, four applications of treatments were made in cultivation: tillering, stem elongation, booting, and anthesis (Zadoks - 2.4, 3.2, 4.5, and 6.1, respectively).

**Table 1:** Components from different treatments were applied to control foliar diseases in the TBIO Sintonia wheat variety.

*T	Application stages			
	Tillering	Stem elongation	Booting	Anthesis
1	–	–	–	–
2	ASM	ASM	ASM	ASM
3	AC + PZ + MO	AC + PZ + MO	AC + PZ + MO	AC + PZ + MO
4	AC + PZ + ASM + MO	AC + PZ + ASM + MO	AC + PZ + ASM + MO	AC + PZ + MO
5	AC + FN + MO	AC + FN + MO	AC + PZ + MO	AC + PZ + MO
6	AC + PZ + MO	AC + PZ + MO	AB + PZ + MO	AB + PZ + MO
7	PZ + ASM	AC + PZ + ASM + MO	AB + PZ + ASM + MO	AB + PZ + MO
8	AC + PZ + MO + SI	AC + PZ + MO + SI	AC + PZ + MO + SI	AC + PZ + MO + SI

*T: treatments; ASM: acibenzolar S-methyl (25 g ha^−1^); AC: azoxistrobin + ciproconazole (0.3 L ha^−1^), PZ: propiconazole (0.5 L ha^−1^); MO: mineral oil (0.6 L ha^−1^); FN: fenpropimorfe (0.5 L ha^−1^); SI: Si (68%) based foliar fertilizer (0.5 kg ha^−1^); AB: azoxistrobin + benzovindiflupir (0.2 kg ha^−1^). A 150 L ha^−1^ of spray volume was applied to each of the treatments.

The gas exchange analysis followed the same procedure as experiment 1; however, it was conducted only once during grain filling and early milk (Zadoks - 7.3). The evaluation of Powdery Mildew (*Blumeria graminis* f.sp. tritici), Leaf Rust (*Puccinia recondita* f.sp. tritici), and Brown Spot (*Bipolaris sorokiniana*) severity on leaves also followed the procedures from experiment 1. Three samples composed of five plants of each treatment were collected. The severity evaluation took place on the flag leaf and the leaf immediately below it.

The following agronomic characters were evaluated in 10 plants per replicate per treatment: the number of spikes m^−2^ (NSP), grains per spike (GPS), the mass of thousand grains (MTG, g), and the grain yield (GY, kg ha^−1^) were measured by harvesting the entire plot (4.8 m^2^).

### 2.3 Economic feasibility analysis on disease control methods

Information on grain yield results and the cost of products used for disease control in both experiments were used to process the Benefit/Cost Ratio (BCR) shown in Equation (1). The BCR is used to identify the most effective and economical treatment and express the financial return for each dollar invested, providing an estimate to the farmer about the rate of return should they opt for a specific cultivation system [35].

Eq 1 $$BCR=\frac{Gross\;Return\;\left(US\$\;{ha}^{-1}\right)}{Total\;cost\;of\;treatment\;\left(US\$\;{ha}^{-1}\right)}$$

### 2.4 Statistical analysis

A programming language based on R-Studio with *agricolae* [36], *ExpDes.pt* [37] and *metan* [38] packages were used for the statistical analysis of the experiments, and the package ggplot2 [39] was used to build the charts. The gas exchange data and yield parameters were submitted to the Shapiro-Wilk test and Bartlet test to verify normality and homogeneity of variance, followed by variance analysis (Two-Factor Analysis for the first experiment: Variety * ASM dose) and Tukey average comparison test. Both experiments' incidence and severity of disease grades were submitted to the Friedman test, because they did not meet the parametric test assumptions. Pearson's correlation analysis was realized for the incidence and severity of disease, gas exchange, and yield parameters.

## 3 Results

### 3.1 Experiment 1

**Fig. 1:**
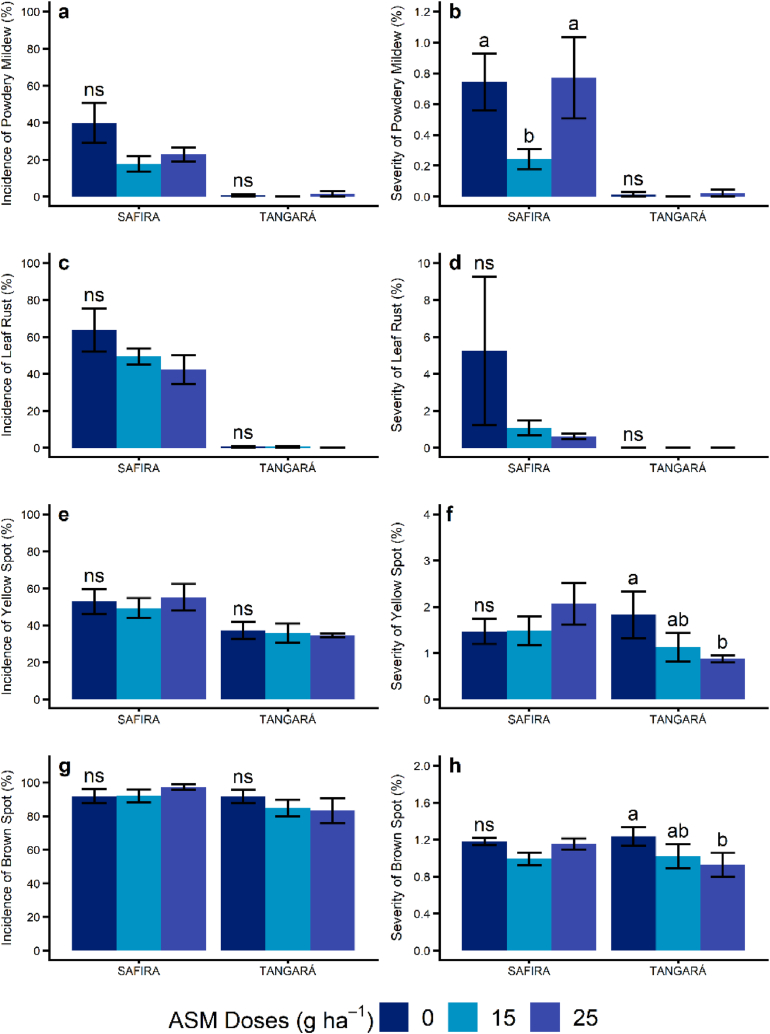
Effect of acibenzolar S-methyl (ASM) doses on the incidence and severity of foliar diseases of Safira and BRS Tangará wheat varieties. a: Incidence of Powdery Mildew; b: Severity of Powdery Mildew; c: Incidence of Leaf Rust; d: Severity of Leaf Rust; e: Incidence of Yellow Spot; f: Severity of Yellow Spot; g: Incidence of Brow Spot; h: Severity of Brow Spot. (*) By Friedman's nonparametric test, means followed by different letters within each variety differ at 5% significance. (^ns^) Not significant. (For interpretation of the references to colour in this figure legend, the reader is referred to the Web version of this article.)

The incidence of Powdery Mildew, Leaf Rust, and Yellow Spot on leaves was higher for the Safira variety, which is a more susceptible genotype for these diseases compared to variety BRS Tangará (Fig. 1a, c, and 1e). Brown Spot incidence was high in both varieties, with no significant difference (Fig. 1g). A high incidence indicates a scenario of high disease pressure, and under these conditions, the BRS Tangará considered moderately resistant to disease did not stand out.

The application of ASM (15 e 25 g ha^−1^) with fungicide reduced the incidence of Powdery Mildew; however, only the smallest dose reduced the severity of the disease compared to the control unit (Fig. 1a and b). For all other diseases evaluated in this study, the combined application of ASM and fungicide did not reduce the disease incidence on the varieties (Fig. 1c, e, and 1g). On the other hand, the combination of 25 g ha^−1^ of ASM and fungicide reduced Leaf Rust on the variety Safira and Yellow Spot and Brown Spot on variety BRS Tangará's leaves in relation to the control groups where only the fungicide was applied (Fig. 1d, f, and 1h).

**Fig. 2:**
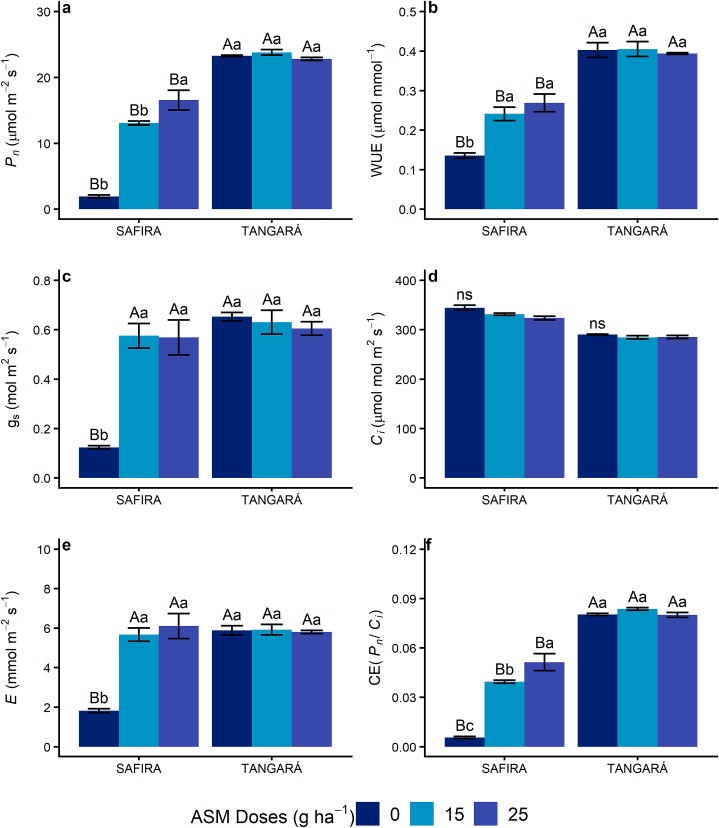
Effect of application of different doses of acibenzolar-S-methyl (ASM) in foliar gas exchange of Safira and BRS Tangará wheat varieties, in Pato Branco, Paraná, Brazil. a: CO_2_ net assimilation rate (*P*_*n*_); b: water use efficiency (WUE); c: stomatal conductance (*gs*); d: intercellular CO_2_ concentration (*C*_*i*_); e: transpiration (E); f: carboxylation efficiency (*CE*=*P*_*n*_/*C*_*i*_). The first application was in tillering (Zadoks – 2.4) and the second was in booting (Zadoks - 4.5). The evaluation was carried out at grain filling, early milk stage (Zadoks – 7.3). (*) Lowercase letters compare different doses in the same variety, while capital letters compare the same dose between varieties. Means followed by distinct letters differ at 5% significance by Tukey's test. (^ns^) Not significant.

No statistical significance was evaluated on gas exchange parameters during the first awns visible stage (data not shown). During the grain filling and early milk evaluation, a significant positive effect was observed on the use of ASM for variety Safira, whether the variety BRS Tangará did not show a response to ASM (Fig. 2). On variety Safira, it was observed that the plants which received the ASM doses showed a larger stomatal opening, characterized by the increase in *gs* (Fig. 2c), which increased the water loss (*E*) (Fig. 2e) but allowed for a higher influx of CO_2_ to leaf cells. The increase of *C*_*i*_ in relation to the control group was not observed (Fig. 2d), as the CE and *P*_*n*_ (Fig. 2f and a) from ASM-treated plants were higher than what was shown by the plants in the control group, also resulting in an improved WUE (Fig. 2b), despite the high transpiration.

**Fig. 3:**
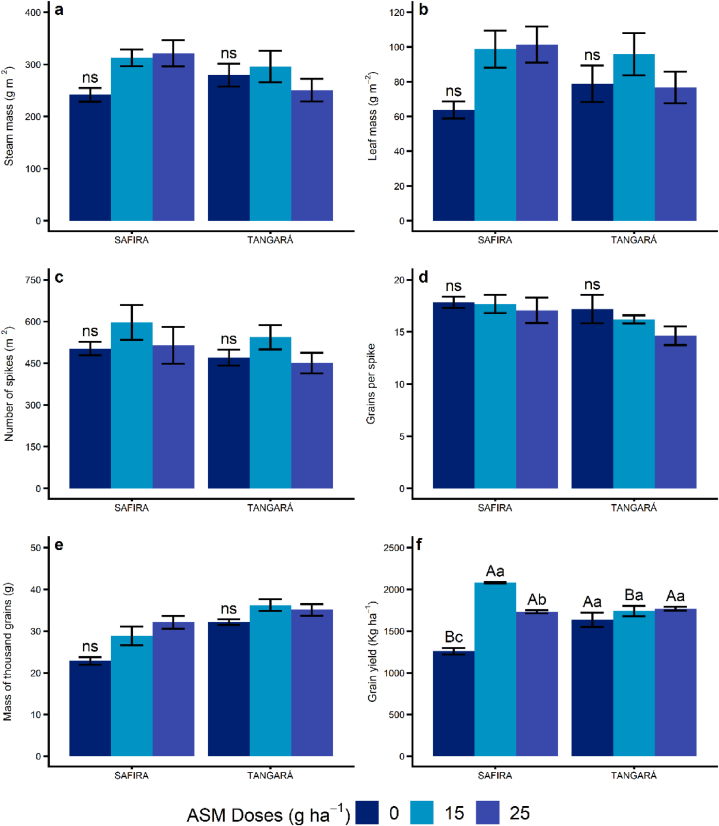
Effect of application of doses of acibenzolar-S-methyl (ASM) on yield components of Safira and BRS Tangará wheat varieties in Pato Branco, Paraná, Brazil. a: steam mass (SM); b: leaf mass (LM); c: number of spikes (NSP); d: grains per spike (GPS); e: mass of thousand grains (MTG); f: grain yield (GY). The first application was in tillering (Zadoks – 2.4), and the second was in booting (Zadoks – 4.5). (*) Lowercase letters compare different doses in the same variety, while capital letters compare the same dose between varieties. Means followed by distinct letters differ at 5% significance by Tukey's test. (^ns^) Not significant.

Considering the standard error, it was observed that using two doses of ASM in the variety Safira promoted an increased accumulation of biomass in stems and leaves compared to the control group (Fig. 3a and b). As for the number of spikes (NSP) and the number of grains per spike (GPS), those were not affected by the use of ASM for any of the tested varieties (Fig. 3c and d). The opposite happened for the mass of thousand grains (MTG), which was higher for both varieties that received the two doses of ASM plus fungicide (Fig. 3e). The higher biomass accumulation in stems, leaves, and grains, induced by the ASM doses in variety Safira, reflected the increase of grain yield when compared to the control group. That way, the grain yield (GY) on that variety was equivalent to the GY of variety BRS Tangará, which is considered more tolerant to diseases, and less responsive to the ASM doses in terms of this variable (Fig. 3f).

The correlation analysis (Table 2) showed that the increase in incidence and severity of foliar diseases, except for Brown Spot (weak correlation), resulted in the reduction of *Pn*, *gs*, *E*, WUE, and CE values, which showed an increase of *Ci*. This relation was more evident for Powdery Mildew and Leaf Rust, which observed the highest correlation coefficients (moderate/strong).

**Table 2:** Pearson correlation between physiological parameters, productive parameters, disease incidence and severity of Safira and BRS Tangará wheat varieties in Pato Branco, Paraná, Brazil.

P^(1)^	*P*_*n*_	*Ci*	*gs*	*E*	WUE	CE	SM	LM	NSP	GPS	MTG	GY
IPM	−0.81*	0.78*	−0.68*	−0.64*	−0.78*	−0.82*	0.07	0.00	0.23	0.33*	−0.61*	−0.45*
SPM	−0.66*	0.68*	−0.57*	−0.42*	−0.70*	−0.69*	0.02	−0.08	0.02	0.18*	−0.46*	−0.37*
ILR	−0.84*	0.89*	−0.59*	−0.54*	−0.86*	−0.87*	0.04	−0.02	0.26	0.42*	−0.70*	−0.17*
SLR	−0.51*	0.42*	−0.50*	−0.50*	−0.46*	−0.49*	−0.33*	−0.35*	−0.13	0.24*	−0.50*	−0.29*
IYS	−0.56*	0.63*	−0.34*	−0.24*	−0.63*	−0.59*	0.12	0.03	0.09	0.20*	−0.42*	−0.10
SYS	−0.13*	0.26*	0.06	0.17*	−0.29*	−0.17*	0.07	0.08	−0.12	0.38*	−0.30*	−0.12*
IBS	−0.24*	0.36*	−0.13*	−0.15*	−0.23*	−0.28*	−0.02	−0.10	−0.10	0.05	−0.26*	−0.09
SBS	−0.14*	0.19*	−0.06	−0.17*	−0.11	−0.16*	−0.04	−0.12*	−0.13	0.42*	−0.42*	−0.42*
*P*_*n*_		−0.91*	0.89*	0.83*	0.95*	0.99*	0.19	0.27*	−0.07	−0.33*	0.80*	0.44*
*Ci*			−0.66*	−0.61*	−0.94*	−0.95*	−0.05	−0.13*	0.14	0.37*	−0.75*	−0.24*
*gs*				0.93*	0.76*	0.84*	0.35	0.40*	0.16	−0.11	0.63*	0.62*
*E*					0.64*	0.78*	0.42*	0.52*	0.13	−0.09	0.64*	0.70*
WUE						0.96*	0.09	0.13	−0.13	−0.42*	0.78*	0.33*
CE							0.15	0.23*	−0.09	−0.35*	0.80*	0.39*
SM								0.91*	0.70*	0.45	0.21	0.45*
LM									0.65*	0.42	0.30	0.47*
NSP										0.41*	0.00	0.23
GPS											−0.54*	−0.14*
MTG												0.44*

*Significance of the Pearson correlation coefficient was at p < 0.05. ^(1)^ P - parameters; IPM and SPM - incidence and severity of powdery mildew, respectively; ILR and SLR - incidence and severity of leaf rust, respectively; IYS and SYS - incidence and severity of yellow spot, respectively; IBS and SBS - incidence and severity of brown spot, respectively; *P*_*n*_ - CO_2_ net assimilation rate; WUE - water use efficiency; *gs* - stomatal conductance; *C*_*i*_ - intercellular CO_2_ concentration; *E* - transpiration; CE - carboxylation efficiency (*P*_*n*_*/C*_*i*_); SM - steam mass; LM - leaf mass; NSP - number of spikes; GPS - grains per spike; MTG - mass of thousand grains; GY - grain yield.

There was a positive correlation between gas exchange parameters (*P*_*n*_, *gs*, *E*, WUE, and CE) x MTG (Table 2). This way, the decrease in gas exchange parameters, caused by the presence and progression of Powdery Mildew, Leaf Rust, and Yellow Spot, has a negative impact on the grain mass accumulation. This response was reinforced by the inverse correlation between the incidence/severity of these diseases x MTG (r > −0.42).

The MTG displayed a positive correlation with GY (r > 0.63, Table 2). This way, by the correlations observed, it became evident that the reduction of Powdery Mildew and Leaf Rust severity, the improvement of gas exchange parameters, and the increase of MTG promoted by the ASM application in variety Safira, had a positive impact on grain yield, characterizing this as an important strategy to be used on corn genotypes with low foliar disease tolerance.

### 3.2 Experiment 2

**Fig. 4:**
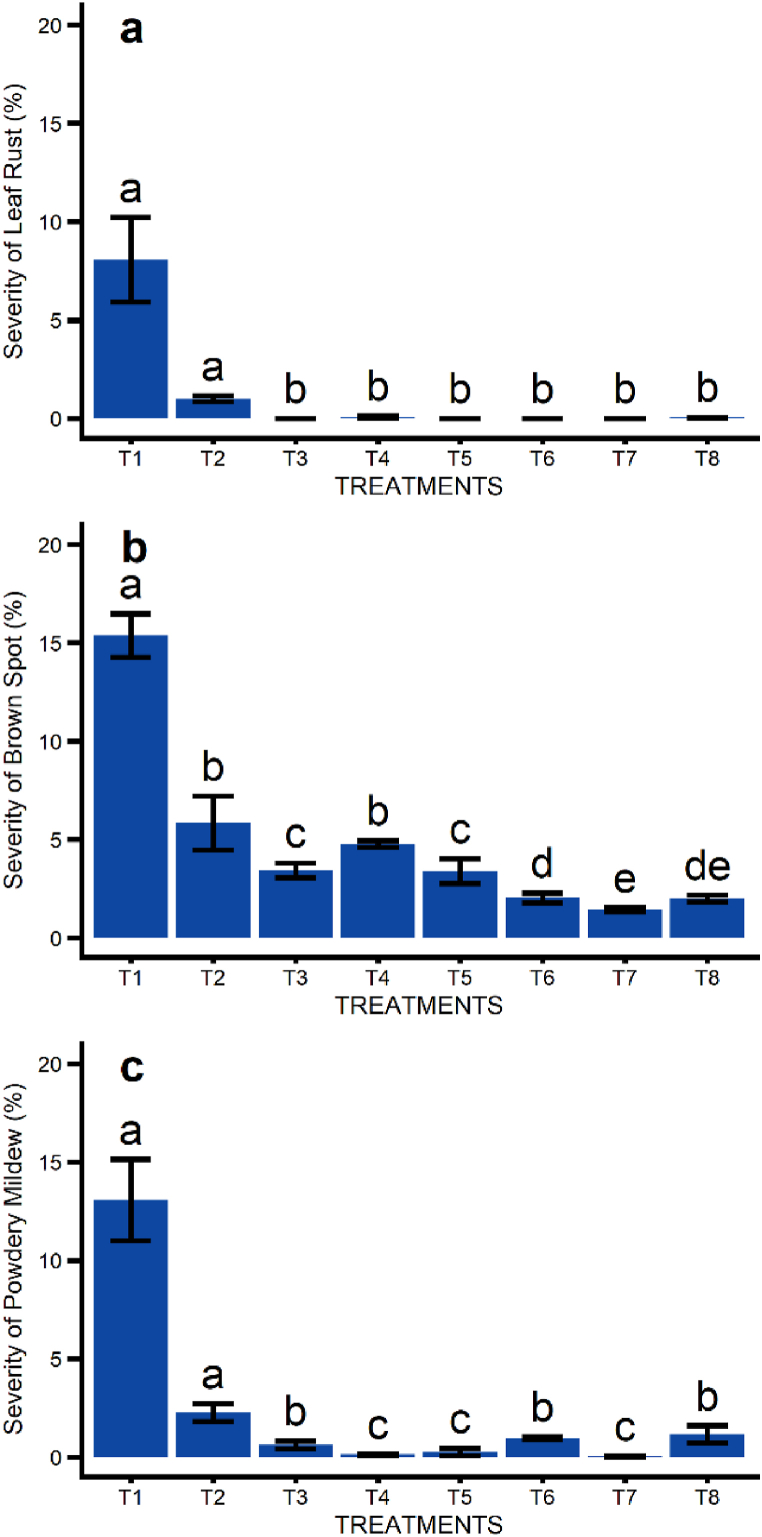
Effect of treatments on Leaf Rust severity (a), Brow Spot severity (b), and Powdery Mildew severity (c) on wheat (TBIO Sintonia) in Passo Fundo, Rio Grande do Sul, Brazil. Applications in tillering, stem elongation, booting, and anthesis (Zadoks - 2.4, 3.2, 4.5, and 6.1). T1: control; T2: ASM (acibenzolar-S-methyl, 25 g ha^−1^) at all stages; T3: AC (azoxistrobin + cyproconazole, 0.3 L ha^−1^) + PZ (propiconazole, 0.5 L ha^−1^) + MO (mineral oil, 0.6 L ha^−1^) at all stages; T4: AC + PZ + MO + ASM in stages 2.4, 3.2, 4.5; AC + PZ + MO in stage 6.1; T5: AC + FN (fenpropimorfe, 0.5 L ha^−1^) + MO in stages 2.4, 3.2; AC + PZ + MO in stages 4.5, 6.1; T6: AC + PZ + MO in stages 2.4, 3.2; AB (azoxistrobin + benzovindiflupir, 0.2 kg ha^−1^) + PZ + MO in stages 4.5, 6.1; T7: PZ + ASM in stage 2.4; AC + PZ + ASM + MO in stage 3.2; AB + PZ + ASM + MO in stage 4.5; AB + PZ + MO in stage 6.1; T8: AC + PZ + SI (Si based foliar fertilizer, 0.5 kg ha^−1^) at all stages. (*) By Friedman's nonparametric test, means followed by distinct letters differ at 5% significance.

All treatments reduced foliar disease severity compared to the control group (Fig. 4). The isolated use of ASM (25 g ha^−1^) during the four applications [Treatment (T) 2] displayed expressive results toward the reduction of disease severity.

In a specific analysis, the Leaf Rust severity was equally reduced by all fungicide treatments or their association with ASM and Si (Fig. 4a). For Brown Spot, the T6 (AC + PZ + MO in stages 2.4, 3.2; AB + PZ + MO in stages 4.5, 6.1), T7 (PZ + ASM in stage 2.4; AC + PZ + ASM + MO in stage 3.2; AB + PZ + ASM + MO in stage 4.5; AB + PZ + MO in stage 6.1) and T8 (AC + PZ + SI at all stages) were the most efficient in reducing severity (Fig. 4b). Considering Powdery Mildew severity, the most noticeable reduction happened with the application of T4 (AC + PZ + MO + ASM in stages 2.4, 3.2, 4.5; AC + PZ + MO in stage 6.1), T5 (AC + FN + MO in stages 2.4, 3.2; AC + PZ + MO in stages 4.5, 6.1) and T7 (Fig. 4c). The T7 (association of fungicides and ASM) was the most effective in severity reduction for all diseases evaluated on this study (Fig. 4).

**Fig. 5:**
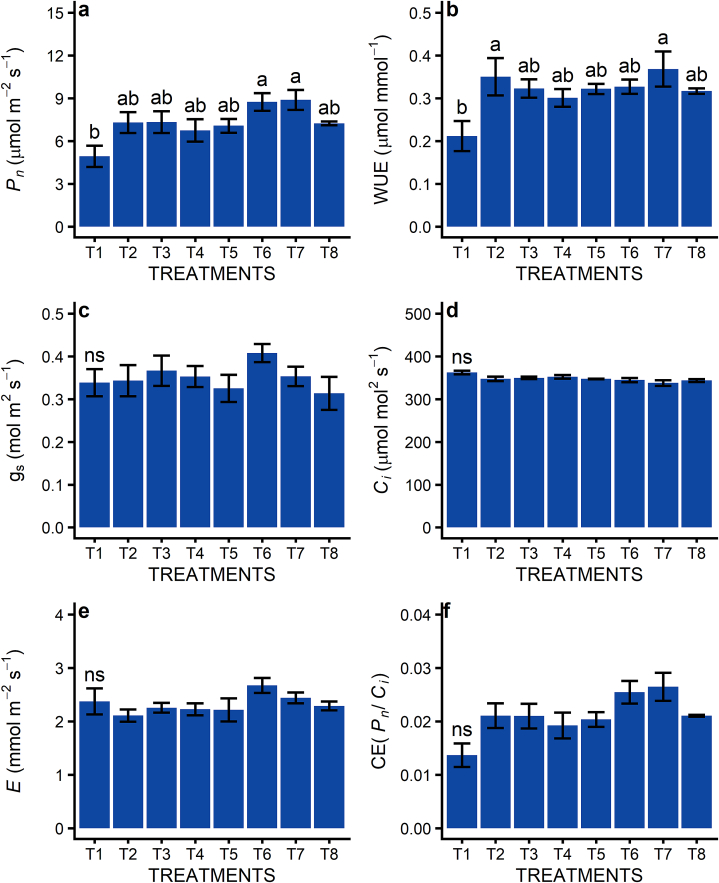
Effect of disease control treatments on foliar gas exchange of wheat (TBIO Sintonia) in Passo Fundo, Rio Grande do Sul, Brazil. a: CO_2_ net assimilation rate (*P*_*n*_); b: water use efficiency (WUE); *c*: stomatal conductance (*gs*); d: intercellular CO_2_ concentration (*C*_*i*_); e: transpiration (E); f: carboxylation efficiency (*CE*=*P*_*n*_/*C*_*i*_). Applications in tillering, stem elongation, booting, and anthesis (Zadoks - 2.4, 3.2, 4.5, and 6.1). The evaluation was carried out at grain filling, early milk stage (Zadoks - 7.3). T1: control; T2: ASM (acibenzolar-S-methyl, 25 g ha^−1^) at all stages; T3: AC (azoxistrobin + cyproconazole, 0.3 L ha^−1^) + PZ (propiconazole, 0.5 L ha^−1^) + MO (mineral oil, 0.6 L ha^−1^) at all stages; T4: AC + PZ + MO + ASM in stages 2.4, 3.2, 4.5; AC + PZ + MO in stage 6.1; T5: AC + FN (fenpropimorfe, 0.5 L ha^−1^) + MO in stages 2.4, 3.2; AC + PZ + MO in stages 4.5, 6.1; T6: AC + PZ + MO in stages 2.4, 3.2; AB (azoxistrobin + benzovindiflupir, 0.2 kg ha^−1^) + PZ + MO in stages 4.5, 6.1; T7: PZ + ASM in stage 2.4; AC + PZ + ASM + MO in stage 3.2; AB + PZ + ASM + MO in stage 4.5; AB + PZ + MO in stage 6.1; T8: AC + PZ + SI (Si based foliar fertilizer, 0.5 kg ha^−1^) at all stages. Means followed by distinct letters differ at 5% significance by Tukey's. (^ns^) Not significant.

Pertaining to the evaluation of gas exchange, it was observed that the application of treatments resulted in an increase of *P*_*n*_ (Fig. 5a) and WUE (Fig. 5b) when compared to the control group, with emphasis on T6 and T7 (*P*_*n*_), T2 (ASM at all stages) and T7 (WUE). On the other hand, there were no differences between the control group and the other treatments for the other parameters evaluated (Fig. 5c, d, 5e and 5f).

**Fig. 6:**
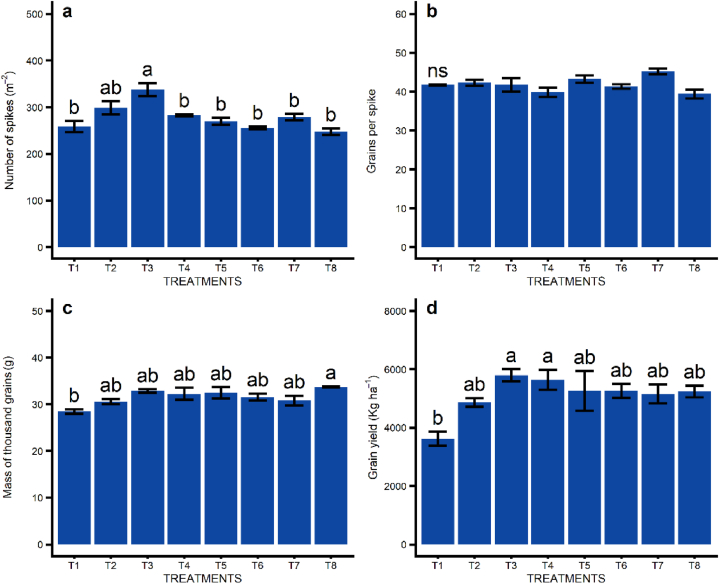
Effect of disease control treatments on wheat yield components (TBIO Sintonia) in Passo Fundo, Rio Grande do Sul, Brazil. a: number of spikes (NSP); b: grains per spike (GPS); c: mass of thousand grains (MTG); d: grain yield (GY). Applications in tillering, stem elongation, booting, and anthesis (Zadoks - 2.4, 3.2, 4.5, and 6.1). T1: control; T2: ASM (acibenzolar-S-methyl, 25 g ha^−1^) at all stages; T3: AC (azoxistrobin + cyproconazole, 0.3 L ha-1) + PZ (propiconazole, 0.5 L ha^−1^) + MO (mineral oil, 0.6 L ha^−1^) at all stages; T4: AC + PZ + MO + ASM in stages 2.4, 3.2, 4.5; AC + PZ + MO in stage 6.1; T5: AC + FN (fenpropimorfe, 0.5 L ha^−1^) + MO in stages 2.4, 3.2; AC + PZ + MO in stages 4.5, 6.1; T6: AC + PZ + MO in stages 2.4, 3.2; AB (azoxistrobin + benzovindiflupir, 0.2 kg ha^−1^) + PZ + MO in stages 4.5, 6.1; T7: PZ + ASM in stage 2.4; AC + PZ + ASM + MO in stage 3.2; AB + PZ + ASM + MO in stage 4.5; AB + PZ + MO in stage 6.1; T8: AC + PZ + SI (Si based foliar fertilizer, 0.5 kg ha^−1^) at all stages. Means followed by distinct letters differ by Tukey's test at 5% significance. (ns) Not significant.

Regarding grain yield and its components, differences were observed between the treatments for NSP, MTG, and GY (Fig. 6a, c, and 6d, respectively). The NSP (Fig. 6b) was superior to the control group on T3 (AC + PZ + MO at all stages), which had no statistical difference from T2. The mass of thousand grains was superior to the control group for T8, without significant differences from the other treatments. For yield analysis, T3 and T4 were superior to the control group. The other treatments did not differ statistically from T3 and T4 and by analyzing the standard error, it is possible to verify that the same promoted higher grain yield compared to the control group.

By correlation analysis, it was observed that the impact of the severity of diseases over the gas exchange parameters and grain production followed the response observed in experiment 1, even with distinct varieties and edaphoclimatic conditions (Table 3). The increase in foliar disease severity caused the reduction of *P*_*n*_, WUE, and CE (r > −0.45) and an increase in *Ci* (r > 0.47).

**Table 3:** Pearson correlation between physiological parameters, productive parameters, and disease severity of TBIO Sintonia wheat variety in Passo Fundo, Rio Grande do Sul, Brazil.

P^(1)^	SPM	SLR	SBS	*P*_*n*_	*Ci*	*gs*	*E*	WUE	CE	NSP	GPS	MTG	GY
SPM	1	0.89*	0.92*	−0.45*	0.47*	0.03	0.11	−0.63*	−0.45*	−0.22	−0.05	−0.55*	−0.70*
SLR		1	0.90*	−0.52*	0.53*	0.05	0.23	−0.66*	−0.51*	−0.10	−0.01	−0.49*	−0.57*
SBS			1	−0.55*	0.59*	0.02	0.03	−0.64*	−0.55*	−0.05	−0.06	−0.60*	−0.64*
*P*_*n*_				1	−0.82*	0.35	0.36	0.82*	0.95*	0.04	0.19	0.23*	0.24*
*Ci*					1	0.24	0.01	−0.85*	−0.86*	0.04	−0.09	−0.24*	−0.30*
*gs*						1	0.71*	−0.03	0.29	0.13	0.16	0.05	−0.05
*E*							1	−0.17	0.33	−0.22	−0.02	0.05	−0.19
WUE								1	0.84*	0.25	0.24	0.26*	0.46*
CE									1	0.02	0.19	0.23*	0.25*
NSP										1	0.27	0.05	0.36*
GPS											1	−0.14	0.05
MTG												1	0.56*

*Significance of the Pearson correlation coefficient was at p < 0.05. ^(1)^ P - parameters; SPM - severity of powdery mildew; SLR - severity of leaf rust; SBS - severity of brown spot; *P*_*n*_ - CO_2_ net assimilation rate; WUE - water use efficiency; *gs* - stomatal conductance; *C*_*i*_ - intercellular CO_2_ concentration; *E* - transpiration; CE - carboxylation efficiency (*P*_*n*_*/C*_*i*_); NSP - number of spikes; GPS - grains per spike; MTG - mass of thousand grains; GY - grain yield.

Inverse correlation between disease severity x MTG (r > −0.49) and between disease severity x GY (r > −0.57) were also observed. As was observed, a direct correlation between MTG and GY (r = 0.56) (Table 3). In this sense, applying of the treatments causes a reduction in disease severity and, consequently, an improvement in photosynthetic performance, and an increase in grain production. This response was also shown in treatments with an exclusive application of ASM, displaying its effectiveness in controlling the evaluated diseases.

### 3.3 Economic analysis

Although the ASM had been effective in increasing the grain production on variety Safira, the economic analysis showed an increase in costs driven by the use of the product, being a limiting factor to the incorporation of this resistance inducer on the wheat disease management control in Pato Branco (Table 4). Within the edaphoclimatic conditions of that place, the increase in grain yield promoted by the use of ASM did not reach a sufficient scale to determine a superior economic return over the treatments using exclusively the fungicide, which presented the highest BCR (23.72).

**Table 4:** Grain yield, gross income, product cost and benefit/cost ratio (BCR) of treatments to control foliar diseases in wheat varieties in Pato Branco, Paraná, Brazil.

Treatments*	Grain yield (Kg ha^−1^)	Gross return** (US$ ha^−1^)	Cost (US$ ha^−1^)	BCR
Safira - fungicide	1260.23	550.62	23.21	23.72
Safira - fungicide + 15 g de ASM	2078.06	907.95	48.11	18.87
Safira - fungicide + 25 g de ASM	1730.30	756.01	64.71	11.68
BRS Tangara - fungicide	1636.17	714.88	23.21	30.80
BRS Tangará - fungicide + 15 g de ASM	1740.59	760.50	48.11	15.80
BRS Tangará - fungicide + 25 g de ASM	1768.02	772.48	64.71	11.94

**Two applications of fungicide (propiconazole in tillering; azoxystrobin + cyproconazole in booting) + ASM, associated with mineral oil. **Wheat price on 05/23/2022: US$ 436.92 a ton. The dollar exchange rate on 05/23/2022 was 1 US$ equals R$ 4.80 (Brazilian real).

For the variety BRS Tangará, resistant to the diseases analyzed, the use of ASM doses for disease control was not justifiable, as the use of the product showed no expressive benefits neither on physiology nor grain yield. The only impact shown was increased production costs and low BCR values (15.80 and 11.94).

In the second experiment, with a susceptible variety TBIO Sintonia, it was observed that the grain yield for the treatments using an association of ASM and chemical control (T4 and T7) was similar to the observed in the treatments with an exclusive fungicide's combination (Table 5). Adding ASM to the control management increased the production costs and resulted in low BCR (14.83 and 13.26, respectively). However, when utilizing ASM by itself (T2), the treatment presented a significantly positive performance (Table 5). The induced plant resistance promoted by ASM and, consequently, the reduction in photosynthesis limitation decurrent from the reduction of disease severity promoted a yield increase of 34% compared with the control group. Although this increase is still lower than the other treatments, the low cost of this disease control strategy promoted a better economic return when it comes to the cultivation conditions in Passo Fundo (BCR = 24.62) (Table 5).

**Table 5:** Grain yield, gross income, product cost, and benefit/cost ratio (BCR) of treatments to control foliar diseases in wheat varieties in Passo Fundo, Rio Grande do Sul, Brazil.

Treatments*	Grain yield (Kg ha^−1^)	Gross return (US$ ha^−1^)**	Cost (US$ ha^−1^)	BCR
1	3623.61	1583.23	0.00	–
2	4867.03	2043.74	83.00	24.62
3	5794.57	2335.92	93.43	25.00
4	5641.57	2309.41	155.68	14.83
5	5259.95	2181.49	127.31	18.70
6	5258.50	2185.39	112.16	19,45
7	5158.42	2095.91	158.08	13.26
8	5236.97	2161.35	126.80	17.04

*T1: control; T2: ASM (acibenzolar-S-methyl, 25 g ha^−1^) at all stages; T3: AC (azoxistrobin + cyproconazole, 0.3 L ha^−1^) + PZ (propiconazole, 0.5 L ha^−1^) + MO (mineral oil, 0.6 L ha^−1^) at all stages; T4: AC + PZ + MO + ASM in stages 2.4, 3.2, 4.5; AC + PZ + MO in stage 6.1; T5: AC + FN (fenpropimorfe, 0.5 L ha^−1^) + MO in stages 2.4, 3.2; AC + PZ + MO in stages 4.5, 6.1; T6: AC + PZ + MO in stages 2.4, 3.2; AB (azoxistrobin + benzovindiflupir, 0.2 kg ha^−1^) + PZ + MO in stages 4.5, 6.1; T7: PZ + ASM in stage 2.4; AC + PZ + ASM + MO in stage 3.2; AB + PZ + ASM + MO in stage 4.5; AB + PZ + MO in stage 6.1; T8: AC + PZ + SI (Si based foliar fertilizer, 0.5 kg ha^−1^) at all stages. **Wheat price on 05/23/2022: US$ 436.92 a ton. The dollar exchange rate on 05/23/2022 was 1 US$ equals R$ 4.80 (Brazilian real).

It is important to highlight that T3, which consists of the same combination of fungicides (azoxystrobin e cyproconazole, + propiconazole) in all applications, displayed a high grain yield and BCR of 25, the highest amongst all treatments (Table 5). On the other hand, the BCR for the treatment using ASM exclusively (T2) was close to the T3, characterizing the two strategies with the best economic feasibility.

## 4 Discussion

The results of physiological and yield parameters, and the high incidence/severity of diseases evaluated, when correlated (Table 2, Table 3), confirmed the negative impact of foliar damage in the assimilation of CO_2_ and the water use efficiency on wheat plants for the two experiments. In the same way [19], observed that the CO_2_ assimilation in wheat plants was reduced in response to the infection of *Magnaporthe oryzae pathotype Triticum*, a fungus responsible for Bruzone. The foliar diseases can change the stomatal conductance, increase the plant's respiration rate, and affect enzymes related to carbon fixation [10].

In our study, the limitations created by diseases affecting the photosynthetically active foliar area and the capacity to synthesize photoassimilates reflect on the reduced biomass allocated in grains and the decrease in crop yield. This was demonstrated in both experiments by the negative correlations between disease severity X mass of thousand grain and disease severity X grain yield (Table 2, Table 3). The carbon gain impact is a fundamental aspect of foliar diseases in crop yield, as the CO_2_ assimilation by photosynthesis creates compounds used in plant's development and yield improvement of plants [40]. This way, the reduction in disease severity, such as Powdery Mildew and Leaf Rust in wheat, promoted by the use of fungicides and ASM, had a positive relationship with the grain yield for the more sensible varieties tested [41]. Also found increased photosynthetic activity and yield increase in strawberries when plants were treated with the elicitors ASM and harpine compared with the control group.

The interaction between the pathogen and host is affected by several factors. According to Ref. [42], the disease development depends on the host's genetics, favorable environmental conditions, the fungal species prevalence, and the survival and dissemination of the pathogen.

Ref. [43] related that the interaction between wheat and *Puccinia recondita*, the fungi responsible for Leaf Rust, depends on temperature, avirulence's genetics, and the interaction between the pathogen's resistance genes, the host's development stage, and its genetic base. According to Ref. [44], leaf rust can cause yield loss in wheat from 7 to 50%, depending on the development stage when the infection occurs. Powdery Mildew is also a fungal disease of great importance, which is disseminated in wheat cultivation areas, registering yield loss of 35, 40, and 62% in Russia, China, and Brazil, respectively [45].

Resistance to Powdery Mildew and Leaf Rust in variety BRS Tangará was confirmed in this study. As per leaf spots, to which the resistance is considered moderate, the use of 25 g ha^−1^ of ASM associated with the fungicide promoted a reduction in the severity of the diseases. We observed the most expressive results on ASM use for varieties that are less tolerant to leaf diseases, Safira and TBIO Sintonia, which displayed reduced severity for Powdery Mildew and Leaf Rust in response to the product. The variety holder classifies variety Safira as moderately resistant to Leaf Rust and Powdery Mildew. However, mutations may have occurred due to being in the Brazilian market for over 15 years, and new resistance genes could be triggered in the pathogen population in response to the selective pressure.

In experiment 1, applying two doses of ASM promoted an increase in stomatal opening and influx of CO_2_ to foliar cells, inevitably increasing transpiration. This chain reaction increased the efficiency of rubisco carboxylation and, consequently, increased the assimilation of CO_2_ in plants treated with ASM, resulting in higher water use efficiency. Therefore, it is noticeable that photosynthesis was not benefited only by the ASM effect in keeping the foliar area healthy but also by the relationship of this response with the stomatal conductance.

Several physiologic processes happen in plants due to pathogen infection, such as the closure of stomata, the hardening of cell walls, modulation in water transport, redistribution of photoassimilates, programmed cellular death, modulation in metal balance, hypertrophy, hyperplasia, among others [12]. The stomata serve as a pathogen entryway to the leaves; that way, it works as a line of defense against infections [46] and, therefore, the control of the fungi responsible for Powdery Mildew and Leaf Rust due to the application of ASM on variety Safira had a direct relationship with the larger stomatal opening (negative correlations between severity x *gs*, Table 2) and the facilitated absorption of CO_2_.

In experiment 2, the increase in photosynthesis and water use efficiency happened similarly in response to all control strategies, including the isolated use of ASM. This way, the increase in CO_2_ assimilation due to foliar tissue preservation and its photosynthetic machinery in treatments with ASM and fungicides promoted an increase in water use efficiency (*P*_*n*_ x WUE, r = 0.82, Table 3).

The reduction in Powderly Mildew and Lead Rust severity by the association of ASM and fungicide in experiment 1 allows us to deduce that inducing the resistance by using an elicitor was an additional mechanism associated with the chemical control, with positive reflex in physiologic and yield parameters. The reduction in foliar damage and increase in photosynthetic performance due to ASM allowed the variety Safira to reach a higher grain yield in relation to the exclusive use of fungicides, equaling its yield to the variety BRS Tangará.

The use of ASM with the fungicide did not influence Powdery Mildew and Leaf Rust severity on variety BRS Tangará and, consequently, its physiological and yield parameters. This response shows that the genetic resistance inherent to the material was enough to control the spread of these diseases. It does not corroborate with the observations of studies by Ref. [30], which suggest that the use of inducers in conditions of low disease pressure results in a reduction in plant's growth and yield once BRS Tangará was treated with ASM and had a similar performance to the control group.

Although plant breeding is the most practical approach to foliar disease management, it is difficult due to sluggishness and the requirement of resistance to multiple diseases [47]. In addition, the enhancement of modern wheat varieties prioritizes high yield and other desired agronomic characteristics, which is associated with the risk of reducing the genetic diversity for disease resistance.

The isolated use of ASM in experiment 2 showed significant results towards the induced resistance as a disease control strategy in wheat. The association of ASM with chemical control did not show significant advantages in disease progression (Fig. 4), gas exchange (Fig. 5), grain yield, and its components (Fig. 6) in comparison with the exclusive use of fungicides. [48], found similar results in wheat research, observing that the addition of ASM to the fungicide (pyraclostrobin + epoxiconazole) did not increase the Powdery Mildew control or yield when compared to the exclusive use of fungicide. On the other hand, using ASM alone reduced Powdery Mildew severity compared to the control group at the same level as when treated with fungicide.

A large number of applications along the production cycle in experiment 2 and the ample diversity of active ingredients used in its fungicide treatments dramatically reduced the disease, making it harder to observe the additional effects of induced resistance by ASM. In addition, it is hard to investigate resistance induction in the field due to a wide range of factors that affect the host's response to the treatments [49].

Although, when applied isolated, the ASM could reduce the spread of symptoms from foliar diseases in TBIO Sintonia, indicating its effectiveness in inducing resistance and strengthening wheat plants' immunity. The preservation of healthy leaf tissue promoted the assimilation of CO_2_ in the flag leaf, and plants that induced resistance were the control strategy applied, reaching yield levels similar to the ones found in treatments based on chemical control.

The induced resistance by ASM, disease protection, and wheat yield increase were observed in other studies. One of the first studies of this kind in wheat was conducted by Ref. [50]. The authors observed that ASM induced SAR in wheat, protecting the plants against Powdery Mildew in the field [51]. had significant results with ASM applied in the field towards the reduction of the Helmintosporiose's (*Drechslera sorokiniana*) area under the disease progress curve (AUDPC) [48]. observed that the use of ASM displayed a significant effect on Leaf Rust control and Powdery Mildew in wheat, as well as an increase in grain yield.

In vase experiments conducted by Ref. [52] confirmed the effectiveness of ASM against *Fusarium graminearum* in wheat, finding that different doses showed different effectiveness depending on the development stage of the plants [28]. observed that the use of 0.075 g L^−1^ associated with *Papiliotrema flavescens* during the anthesis in wheat displayed the best performance in reducing Fusarium head blight's severity. The authors also determined that ASM high doses performed better during anthesis, whilst smaller doses were more effective when applied before the late boot stage.

Disease control strategies should allow the analysis of its efficiency in keeping the plant healthy and the reflex on its physiology and grain yield. It is fundamental to consider the net income generated by the application of the technology in order to choose an economically feasible option.

In our experiments, we verified that the association of ASM with fungicide in the disease control in susceptible wheat varieties, even when the treatment is effective in reducing Powdery Mildew and Leaf Rust, does not promote a yield increase sufficient to reach an economic return superior to exclusive chemical control (Table 4).

However, the results related to the isolated use of ASM (experiment 2) reveal not only its effect on the evaluated disease control but also a subtle and positive impact on grain yield (Table 5). Even though the yield increment was inferior to the ones from the chemical treatments, the fact that there was no use of fungicides helped significantly reduce the control costs, making the isolated use of ASM an economically viable strategy due to its benefit/cost ratio. With its BCR's value being very close to the Azoxystrobin, and Cyproconazole + Propiconazole in four applications (representing the highest financial return amongst all the treatments), the usage of ASM showed promising by its efficiency, economy and the ability to avoid constant use of the same fungicide's active ingredient in wheat disease control [53]. suggest that the mix of ASM with fungicides can be a promising disease control strategy and reduce the chances of fungi resistance to fungicides.

## 5 Conclusion

This study showed the limitations of incorporating ASM into foliar fungal disease control in economic terms. However, it was evident that ASM effectively induced plant resistance against Leaf Rust and Powdery Mildew in the field, and can be considered a sustainable option for wheat cultivation, reducing dependence on synthetic fungicides. Even though its association with chemical control was not the best economic strategy, the use of ASM is a tool that can be incorporated into wheat cultivation to minimize the emergence of fungicide-resistant pathogens due to the diversification of modes of action employed and reduce the toxic residue deposition to the environment and human health (see Table 5, treatments T2 and T3).

To allow the implementation on a large scale as a new management option for farmers, it is important to realize more investigations to explore different doses and application technologies of ASM in the field, where the incidence and severity of diseases are widely variable due to climatic conditions.

## Funding

MBR (Syngenta Crop Protection) made the ASM available for the experiments. LVD received a doctoral scholarship from Coordination for the Improvement of Higher Education Personnel (10.13039/501100002322CAPES) finance code 001, and MAB, DC, and MVP received an undergraduate scholarship from National Council for Scientific and Technological Development (10.13039/501100003593CNPq, Brazil).

## Author contributions

1) conceived and designed the experiments: Mateus Batista Remor and José Abramo Marchese.

2) performed the experiments: Marco Antônio Bosse, Diogo Capelin, Marcos Vily Paladini, Mateus Batista Remor and José Abramo Marchese.

3) analyzed and interpreted the data: Lucas Vinicius Dallacorte, José Donizetti de Lima, Felipe Cattani, Anelise Tessari Perboni and José Abramo Marchese.

4) contributed reagents, materials, analysis tools or data: Mateus Batista Remor and José Abramo Marchese.

5) wrote the paper: Lucas Vinicius Dallacorte, Felipe Cattani, Anelise Tessari Perboni and José Abramo Marchese.

## Declaration of competing interest

The authors declare the following financial interests/personal relationships which may be considered as potential competing interests:Mateus Batista Remor reports supplies was provided by Syngenta Crop Protection. Mateus Batista Remor reports a relationship with Syngenta Crop Protection that includes: employment. The authors declare that Mateus Batista Remor (co-author), a collaborator at Syngenta Crop Protection, made the ASM product available for application in the experiments and participated in the conceptualization, definition of methodology and conduct of one of the experiments.
