# Supplementary material for: Economic versus technical efficiency in using ASM combined with fungicides to elicit wheat varieties with different disease susceptibilities
Source: Heliyon. 2023 Jun 9;9(6):e17012. doi: 10.1016/j.heliyon.2023.e17012 (PMC10361120; doi:10.1016/j.heliyon.2023.e17012)
Supplement: Multimedia component 1 [file mmc1.docx]

Supplementary material

**Supplementary Figure 1.** Maximum, and minimum temperature, relative humidity, and precipitation accumulation measured during the experiments in Pato Branco, PR, Brazil, obtained from Meteorological Station SIMEPAR/IDR (A), and in Passo Fundo, RS, Brazil, obtained from Meteorological Station 83914, BDMEP/INMET (B).


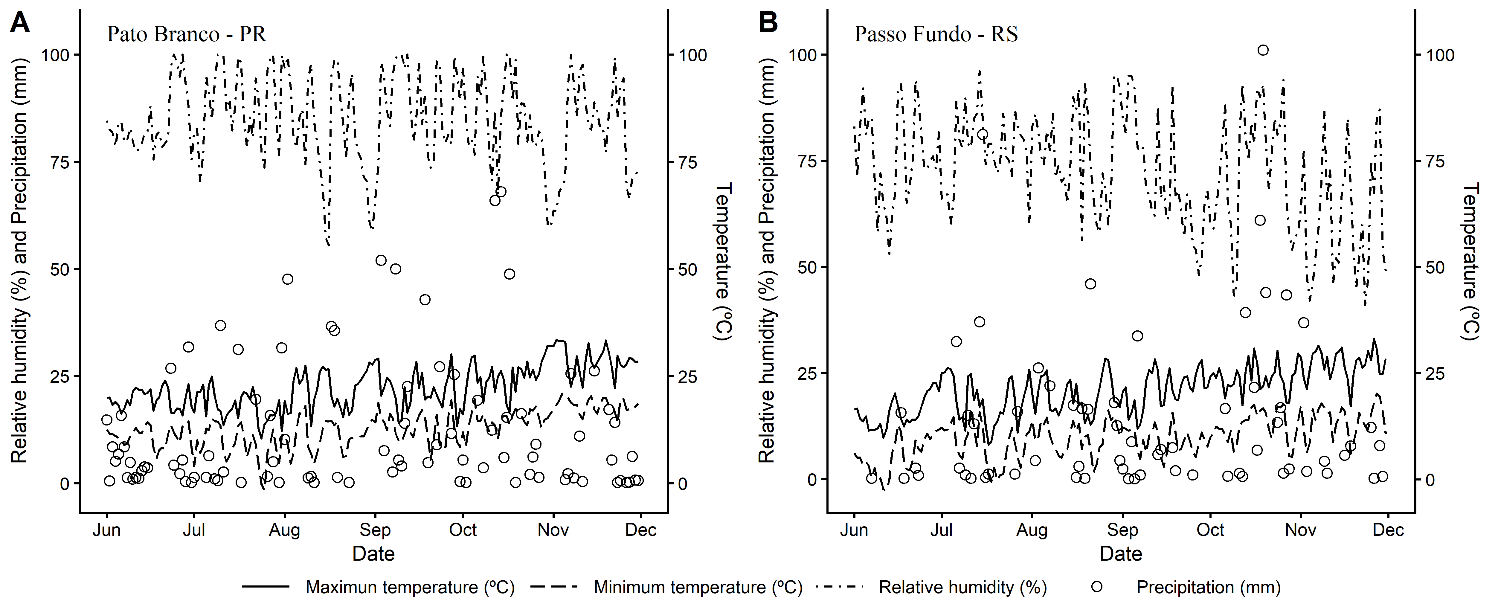


**Supplementary Table 1.** Quotation of products used in experiments for economic feasibility analysis.

| Acronym | Active ingredient | Unity | Cost (US$)* |
| --- | --- | --- | --- |
| AC | Azoxystrobin + Cyproconazole | 1 L | 41.07 |
| PZ | Propiconazole | 1 L | 14.07 |
| MO | Mineral Oil | 1 L | 6.67 |
| ASM | Acibenzolar S-methyl | 1 g | 0.83 |
| SI | Si (68%) based foliar fertilizer | 1 Kg | 16.68 |
| AB | Azoxystrobin a + Benzovindiflupyr | 1 Kg | 108.41 |
| FN | Fenpropimorph | 1 L | 47.95 |

* The dollar exchange rate on 05/23/2022 was 1 US$ equals R$ 4.80 (Brazilian real).

**Supplementary Table 2.** Description of treatments and product costs to control foliar diseases in wheat varieties grown in Pato Branco, Paraná, Brazil.

| Applications | | | | Cost (US$ ha^-1^) |
| --- | --- | --- | --- | --- |
| 1st | | 2nd | |  |
| Active ingredient | Dose | Active ingredient | Dose |  |
| PZ | 0.3 L ha^-1^ | AC | 0.3 L ha^-1^ | 23.21 |
| MO | 0.5 L ha^-1^ | MO | 0.5 L ha^-1^ |  |
| PZ | 0.3 L ha^-1^ | AC | 0.3 L ha^-1^ | 48.11 |
| ASM | 15 g ha^-1^ | ASM | 15 g ha^-1^ |  |
| MO | 0.5 L ha^-1^ | MO | 0.5 L ha^-1^ |  |
| PZ | 0.3 L ha^-1^ | AC | 0.3 L ha^-1^ | 64.71 |
| ASM | 25 g ha^-1^ | ASM | 25 g ha^-1^ |  |
| MO | 0.5 L ha^-1^ | MO | 0.5 L ha^-1^ |  |

PZ: propiconazole; AC: Azoxystrobin + Cyproconazole; ASM: acibenzolar S-methyl; MO: mineral oil. The dollar exchange rate on 05/23/2022 was 1 US$ equals R$ 4.80 (Brazilian real).

**Supplementary Table 3.** Description of treatments and product costs to control foliar diseases in wheat varieties grown in Passo Fundo, Rio Grande do Sul, Brazil.

| T* | Applications | | | | | | | | Cost (US$ ha^-1^) |
| --- | --- | --- | --- | --- | --- | --- | --- | --- | --- |
|  | 1st | | 2nd | | 3th | | 4th | |  |
|  | AI | Dose | AI | Dose | AI | Dose | AI | Dose |  |
| 1 | - | - | - | - | - | - | - | - | - |
| 2 | ASM | 25 g ha^-1^ | ASM | 25 g ha^-1^ | ASM | 25 g ha^-1^ | ASM | 25 g ha^-1^ | 83.00 |
| 3 | AC | 0.3 L ha^-1^ | AC | 0.3 L ha^-1^ | AC | 0.3 L ha^-1^ | AC | 0.3 L ha^-1^ | 93.43 |
|  | PZ | 0.5 L ha^-1^ | PZ | 0.5 L ha^-1^ | PZ | 0.5 L ha^-1^ | PZ | 0.5 L ha^-1^ |  |
|  | MO | 0.6 L ha^-1^ | MO | 0.6 L ha^-1^ | MO | 0.6 L ha^-1^ | MO | 0.6 L ha^-1^ |  |
| 4 | AC | 0.3 L ha^-1^ | AC | 0.3 L ha^-1^ | AC | 0.3 L ha^-1^ | AC | 0.3 L ha^-1^ | 155.68 |
|  | PZ | 0.5 L ha^-1^ | PZ | 0.5 L ha^-1^ | PZ | 0.5 L ha^-1^ | PZ | 0.5 L ha^-1^ |  |
|  | ASM | 25 g ha^-1^ | ASM | 25 g ha^-1^ | ASM | 25 g ha^-1^ | MO | 0.6 L ha^-1^ |  |
|  | MO | 0.6 L ha^-1^ | MO | 0.6 L ha^-1^ | MO | 0.6 L ha^-1^ | - | - |  |
| 5 | AC | 0.3 L ha^-1^ | AC | 0.3 L ha^-1^ | AC | 0.3 L ha^-1^ | AC | 0.3 L ha^-1^ | 127.31 |
|  | FN | 0.5 L ha^-1^ | FN | 0.5 L ha^-1^ | PZ | 0.5 L ha^-1^ | PZ | 0.5 L ha^-1^ |  |
|  | MO | 0.6 L ha^-1^ | MO | 0.6 L ha^-1^ | MO | 0.6 L ha^-1^ | MO | 0.6 L ha^-1^ |  |
| 6 | AC | 0.3 L ha^-1^ | AC | 0.3 L ha^-1^ | AB | 0.2 kg ha^-1^ | AB | 0.2 kg ha^-1^ | 112.16 |
|  | PZ | 0.5 L ha^-1^ | PZ | 0.5 L ha^-1^ | PZ | 0.5 L ha^-1^ | PZ | 0.5 L ha^-1^ |  |
|  | MO | 0.6 L ha^-1^ | MO | 0.6 L ha^-1^ | MO | 0.6 L ha^-1^ | MO | 0.6 L ha^-1^ |  |
| 7 | PZ | 0.5 L ha^-1^ | AC | 0.3 L ha^-1^ | AB | 0.2 kg ha^-1^ | AB | 0.2 kg ha^-1^ | 158.08 |
|  | ASM | 25 g ha^-1^ | PZ | 0.5 L ha^-1^ | PZ | 0.5 L ha^-1^ | PZ | 0.5 L ha^-1^ |  |
|  | - | - | ASM | 25 g ha^-1^ | ASM | 25 g ha^-1^ | MO | 0.6 L ha^-1^ |  |
|  | - | - | MO | 0.6 L ha^-1^ | MO | 0.6 L ha^-1^ | - | - |  |
| 8 | AC | 0.3 L ha^-1^ | AC | 0.3 L ha^-1^ | AC | 0.3 L ha^-1^ | AC | 0.3 L ha^-1^ | 126.79 |
|  | PZ | 0.5 L ha^-1^ | PZ | 0.5 L ha^-1^ | PZ | 0.5 L ha^-1^ | PZ | 0.5 L ha^-1^ |  |
|  | MO | 0.6 L ha^-1^ | MO | 0.6 L ha^-1^ | MO | 0.6 L ha^-1^ | MO | 0.6 L ha^-1^ |  |
|  | SI | 0.5 kg ha^-1^ | SI | 0.5 kg ha^-1^ | SI | 0.5 kg ha^-1^ | SI | 0.5 kg ha^-1^ |  |

*T - treatments; AI - Active ingredient; ASM: acibenzolar S-methyl; AC: Azoxystrobin + Cyproconazole; PZ: propiconazole; MO: mineral oil; FN: Fenpropimorph; AB: Azoxystrobin + Benzovindiflupyr; SI: Si (68%) based foliar fertilizer. The dollar exchange rate on 05/23/2022 was 1 US$ equals R$ 4.80 (Brazilian real).
